# Single‐Molecule Two‐Color Coincidence Detection of Unlabeled alpha‐Synuclein Aggregates

**DOI:** 10.1002/anie.202216771

**Published:** 2023-02-28

**Authors:** Alexandre Chappard, Craig Leighton, Rebecca S. Saleeb, Kiani Jeacock, Sarah R. Ball, Katie Morris, Owen Kantelberg, Ji‐Eun Lee, Elsa Zacco, Annalisa Pastore, Margaret Sunde, David J. Clarke, Patrick Downey, Tilo Kunath, Mathew H. Horrocks

**Affiliations:** ^1^ EaStCHEM School of Chemistry The University of Edinburgh Edinburgh EH9 3FJ UK; ^2^ Centre for Regenerative Medicine Institute for Stem Cell Research School of Biological Sciences The University of Edinburgh Edinburgh EH16 4UU UK; ^3^ School of Medical Sciences Faculty of Medicine and Health, and Sydney Nano The University of Sydney Sydney NSW 2006 Australia; ^4^ Centre for Human Technologies (CHT) Istituto Italiano di Tecnologia (IIT) Via Enrico Melen, 83 16152 Genova Italy; ^5^ European Synchrotron Radiation Facility 71 Ave des Martyrs 38000 Grenoble France; ^6^ UCB Biopharma S.P.R.L. Braine l'Alleud Belgium

**Keywords:** Aggregation or Oligomerization, Fluorescence, Microscopy, Proteins, Single-Molecule

## Abstract

Protein misfolding and aggregation into oligomeric and fibrillar structures is a common feature of many neurogenerative disorders. Single‐molecule techniques have enabled characterization of these lowly abundant, highly heterogeneous protein aggregates, previously inaccessible using ensemble averaging techniques. However, they usually rely on the use of recombinantly‐expressed labeled protein, or on the addition of amyloid stains that are not protein‐specific. To circumvent these challenges, we have made use of a high affinity antibody labeled with orthogonal fluorophores combined with fast‐flow microfluidics and single‐molecule confocal microscopy to specifically detect α‐synuclein, the protein associated with Parkinson's disease. We used this approach to determine the number and size of α‐synuclein aggregates down to picomolar concentrations in biologically relevant samples.

## Graphical abstract

Pathological protein aggregates in neurodegenerative disorders are difficult to characterise using current methods. We present a novel single‐molecule detection method to specifically detect and characterise α‐synuclein aggregates at picomolar concentrations. We demonstrate the ability to detect aggregates in biologically relevant samples.

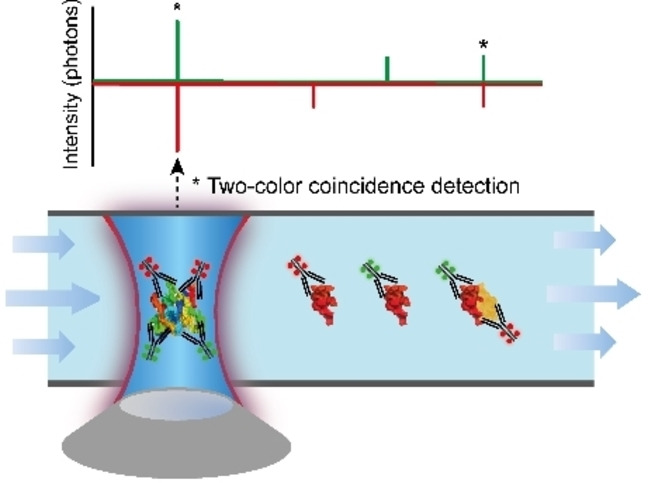

## Introduction

Protein aggregation is a key molecular feature that underlies a multitude of neurodegenerative disorders, including Alzheimer's (AD) and Parkinson's disease (PD). The assembly of alpha‐synuclein (α‐syn), an intrinsically disordered pre‐synaptic protein, into intraneuronal cytoplasmic inclusions, termed Lewy bodies (LBs), is a pathological hallmark of PD.^[1]^ The importance of α‐syn in PD is further evidenced by mutations and multiplications in the *SNCA* gene which encodes α‐syn protein leading to early‐onset familial PD.[^2^, ^3^, ^4^, ^5^, ^6^] Although LB pathology has traditionally been used to define PD, it is the early soluble aggregates, known as oligomers, that have been shown to be the most cytotoxic species,[^7^, ^8^, ^9^, ^10^] and emerging evidence suggests they play a central role in the propagation of pathology throughout the brain (reviewed here Ref. [11]).

Despite their evidenced importance to the disease, their low abundance and high heterogeneity, both in terms of size and structure, has made oligomers difficult to study using traditional biochemical techniques. We have previously used single‐molecule fluorescence techniques to characterize the aggregation and behavior of fluorescently tagged, recombinantly expressed α‐syn, determining both the size and structures of the oligomers formed.[^12^, ^13^] These techniques, however, are limited to studying labeled protein, and do not account for the modifying effects of the tag on protein conformation and aggregation kinetics. The divergent kinetic profiles observed for amyloid beta labeled with five different fluorescent dyes highlights this issue.^[14]^ Furthermore, the overall charge of the protein may be affected by the addition of an extrinsic dye molecule, which could increase adsorption onto surfaces and alter the effective concentration of the protein being studied.^[15]^ Most significantly, these methods are not conducive to monitoring α‐syn aggregation in human biological samples such as cerebrospinal fluid (CSF), where their presence could be used as an early indicator of disease.[^16^, ^17^, ^18^]

As an alternative to using labeled protein, we and others have used the amyloid‐binding dye thioflavin‐T (ThT) with single‐molecule techniques to directly observe individual aggregates in CSF samples.[^16^, ^19^, ^20^] The major drawback of using ThT for detection, however, is its lack of specificity, binding to a common motif of the extended β‐sheet structure present in many amyloid‐related protein aggregates.^[21]^ Moreover, only mature aggregates bind ThT, meaning that earlier, potentially more damaging oligomers cannot be characterized using such an approach. More recently, a range of other amyloid‐binding fluorophores have been developed that have shown promise for identifying earlier aggregates;[^22^, ^23^, ^24^] however, these still lack the ability to distinguish between those formed from different proteins.

Monoclonal antibodies (mAbs) offer an attractive alternative for specifically targeting label‐free proteins‐of‐interest. Non‐specific adsorption onto glass surfaces, however, limits the use of mAbs for direct visualization of protein aggregates at single‐molecule resolution using wide‐field microscopy approaches. Alternatively, single‐molecule confocal microscopy can be used to study fluorescent species in solution. Such an approach, however, is limited to an upper fluorophore concentration of ≈100 pM, meaning that either sub‐nanomolar affinity antibodies are needed, or complex microfluidic devices for rapid dilution are required for detection.[^25^, ^26^]

In this work, we have used a picomolar‐affinity aggregate‐binding antibody developed by UCB Biopharma to directly visualize unlabeled α‐syn using single‐molecule confocal microscopy.^[27]^ By conjugating separate batches of the antibody with either Alexa Fluor 488 (AF488) or Alexa Fluor 647 (AF647) fluorophores, and introducing them to solutions of α‐syn, we were able to distinguish oligomers from monomers using two color coincidence detection (TCCD).^[28]^ We compared our approach with single‐molecule confocal detection using ThT, demonstrating that we were able to detect a >20‐fold higher number of protein aggregates. Finally, we measured aggregates spiked into human CSF at a physiologically relevant concentration to ensure that our technique can accurately detect unlabeled α‐syn aggregates in a complex biological mixture.

## Results and Discussion

### Single‐molecule detection of unlabeled α‐syn aggregates

**Figure 1:**
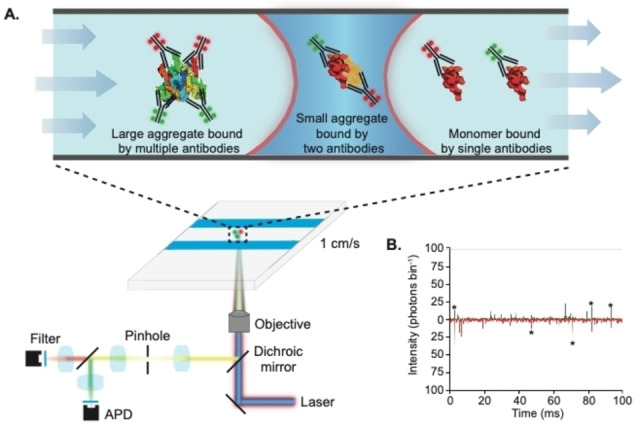
TCCD detection of antibody‐tagged α‐syn aggregates. a) Schematic of the experimental setup. b) Representative TCCD single‐molecule data taken from α‐syn aggregate‐containing solutions, showing intensity from AF488‐tagged antibodies (green) and AF647‐tagged antibodies (red). Stars show example coincident bursts corresponding to protein aggregates.

To specifically detect individual α‐syn aggregates, we labeled batches of the high affinity α‐syn antibody with two orthogonal fluorophores, AF488 and AF647 (see Supporting Information and Figures S2 and S3 for experimental details and determination of label stoichiometry). Since monomers only contain a single binding site for the antibody, they will be bound by either an AF488‐ or AF647‐tagged antibody. Meanwhile, aggregates containing multiple epitopes are likely to have a mix of both labeled antibodies and will therefore give rise to coincident fluorescent bursts as they transit the confocal volume (Figure 1). Whereas we have previously used Förster Resonance Energy Transfer (FRET) with single‐molecule confocal microscopy to detect and characterize directly labeled α‐syn,^[12]^ no FRET was detected between the labeled antibodies.

**Figure 2:**
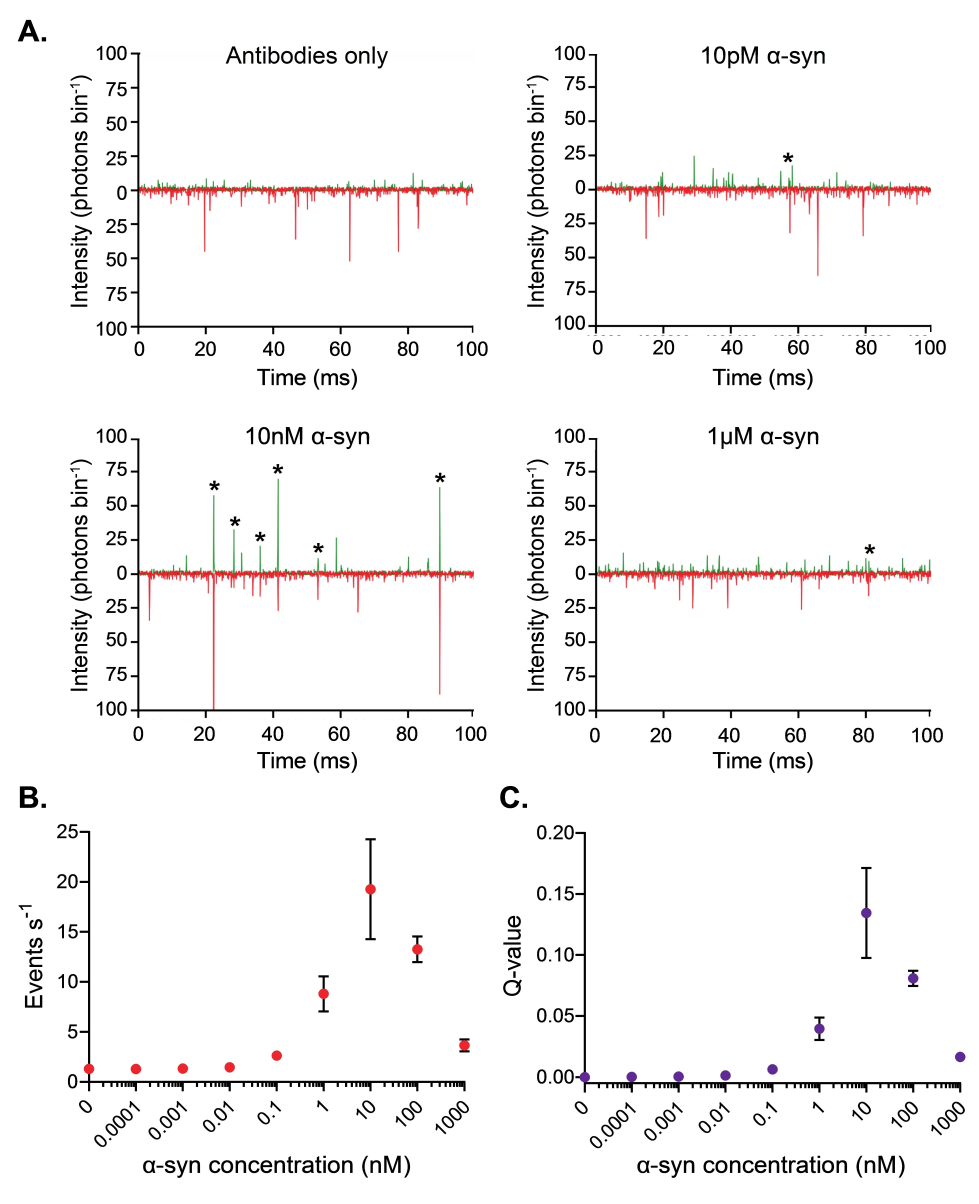
TCCD measurement of a concentration series of unlabeled α‐syn aggregates. a) Representative TCCD data for samples containing 0 pM, 10 pM, 10 nM, and 1 μM of α‐syn subjected to conditions favoring aggregation for 24 h at a higher concentration. Example coincident bursts are starred. b) Plots of coincident event rate per second and c) association quotient *Q* shown for varying concentrations of α‐syn. Data are shown as mean±SD, *n*=3.

We first sought to determine the response of our approach to varying concentrations of α‐syn aggregates. This was achieved by diluting α‐syn aggregates to a series of concentrations spanning several orders of magnitude into a solution containing the optimized antibody concentrations of 20 pM AF488‐ and 20 pM AF647‐tagged antibody (see Supporting Information and Figure S4 for determination of optimum antibody concentration). As expected from its *K*_D_ of 30 pM (see Supporting Information and Table S1), the number of coincident bursts (Figure 2a) is negligible at concentrations of α‐syn below 10 pM, before increasing at higher concentrations. It subsequently decreases at micromolar concentrations. Both the event rate (Figure 2b), defined as the number of coincident events per unit time, and the association quotient, Q, the fraction of coincident events (for further details, see Supporting Information and Figure S1 for a schematic representation of how the Q‐value is calculated, and details of the threshold selection) (Figure 2c) reflect this change, with them both increasing at α‐syn concentrations above 10 pM, before decreasing at α‐syn concentrations higher than 100 nM. This decrease is attributed to the limited antibody present in solution compared to the concentration of α‐syn, resulting in a lower proportion of aggregates being bound by multiple antibodies.

The limit of detection (LoD) is the lowest readout likely to be reliably distinguished from a sample containing no analyte. It can be calculated by determining Q from both a sample containing no α‐syn to determine the limit of blank (LoB), and a sample of α‐syn at a low concentration. For this approach, the LoB had a Q‐value of 5.4×10^−4^, while the LoD had a Q‐value of 8.7×10^−4^, which corresponds to a concentration of ≈10 pM.

The success of the high sensitivity of this approach can be attributed to multiple factors. First, the concentration of the antibody is kept low to ensure that individual molecules can be observed. Second, the affinity of the antibody (picomolar *K*_D_ for aggregates, see Supporting Information, and Table S1) ensures a high proportion of the α‐syn present in aggregates is bound at low concentrations. And finally, fast flow microfluidics ensures that the data acquisition rate is high enough to detect and characterize a high number of events.

### Specificity of the approach to α‐syn oligomers

**Figure 3:**
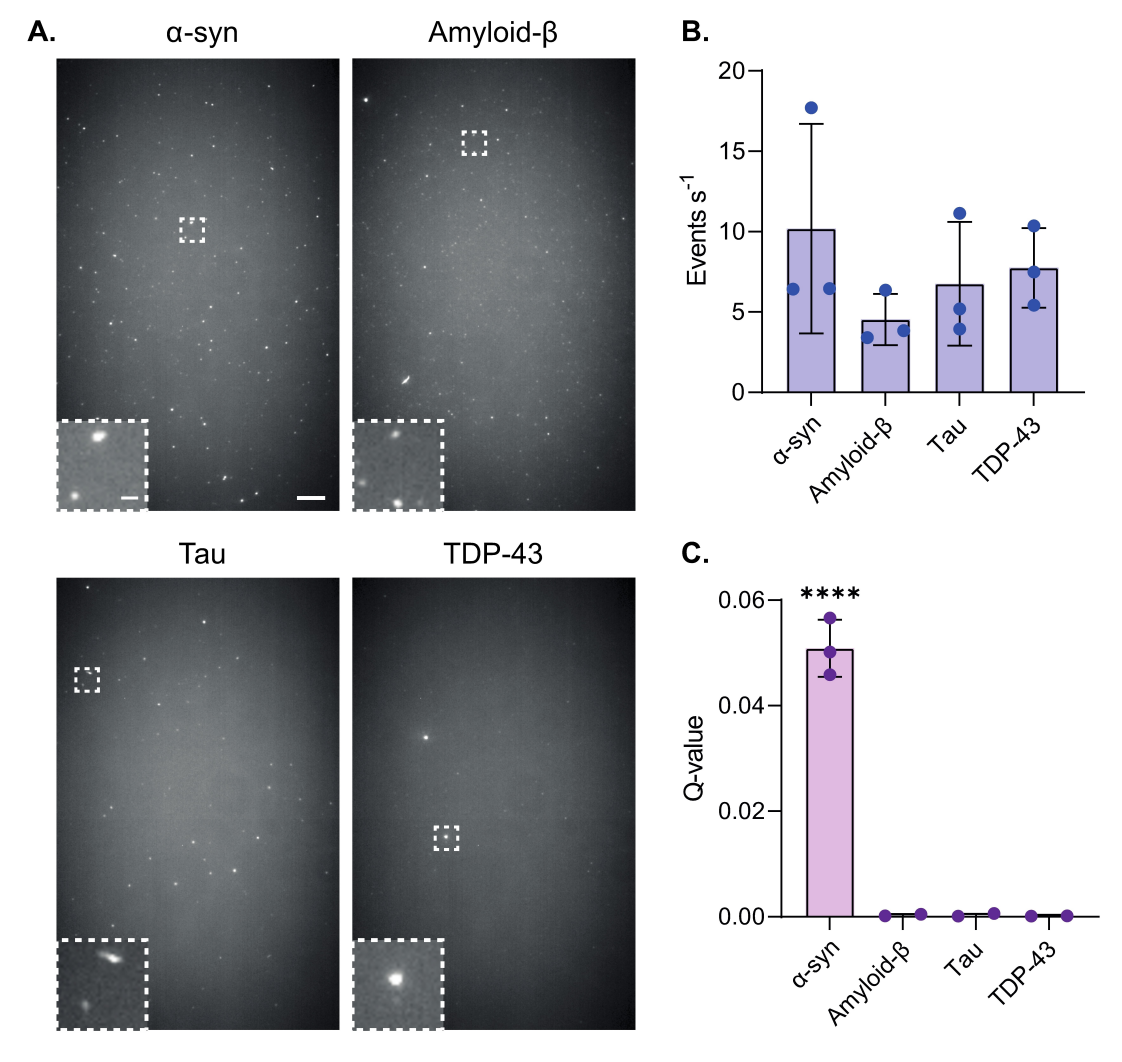
TCCD approach is specific to α‐syn aggregates. a) Representative ThT SAVE images of 500 nM α‐syn, amyloid‐β, tau, and TDP‐43. Scale bars are 5 μm and 1 μm in length for the full field of views and insets, respectively. b) Quantification of ThT‐active species detected for each amyloid protein using single‐molecule confocal microscopy. c) TCCD detection of amyloid proteins. Data shown as mean±SD, *n*=3. **** *P*<0.0001, One‐way ANOVA with Tukey multiple comparisons test.

Previous single‐molecule approaches for detecting unlabeled aggregates using ThT were unable to identify those formed from different proteins.[^16^, ^19^] We therefore set out to determine whether our approach could be used to distinguish α‐syn aggregates from those composed of amyloid‐beta‐1‐42 (Aβ‐42), tau, and a fragment of TDP‐43 containing both RNA Recognition Motifs (RRM1‐2).^[29]^ The number of aggregates in each protein sample was first detected using ThT with both Single Aggregate Visualization by Enhancement (SAVE) imaging^[16]^ (Figure 3a), and single‐molecule confocal microscopy (Figure 3b). As expected, aggregates were detected in all samples using ThT, with a similar event rate observed in each case. Our TCCD approach, however, was only able to detect aggregates formed from α‐syn, thereby demonstrating its specificity and its applicability for detecting higher‐order α‐syn structures in complex mixtures (Figure 3c).

### Determining the kinetics of α‐syn aggregation

Single‐molecule studies combined with kinetic modelling have been used to describe the aggregation process and provide insights into the molecular mechanisms involved in the spread of α‐syn pathology in the brain.[^7^, ^30^] We therefore investigated the ability of our TCCD approach to follow the aggregation of α‐syn.

**Figure 4:**
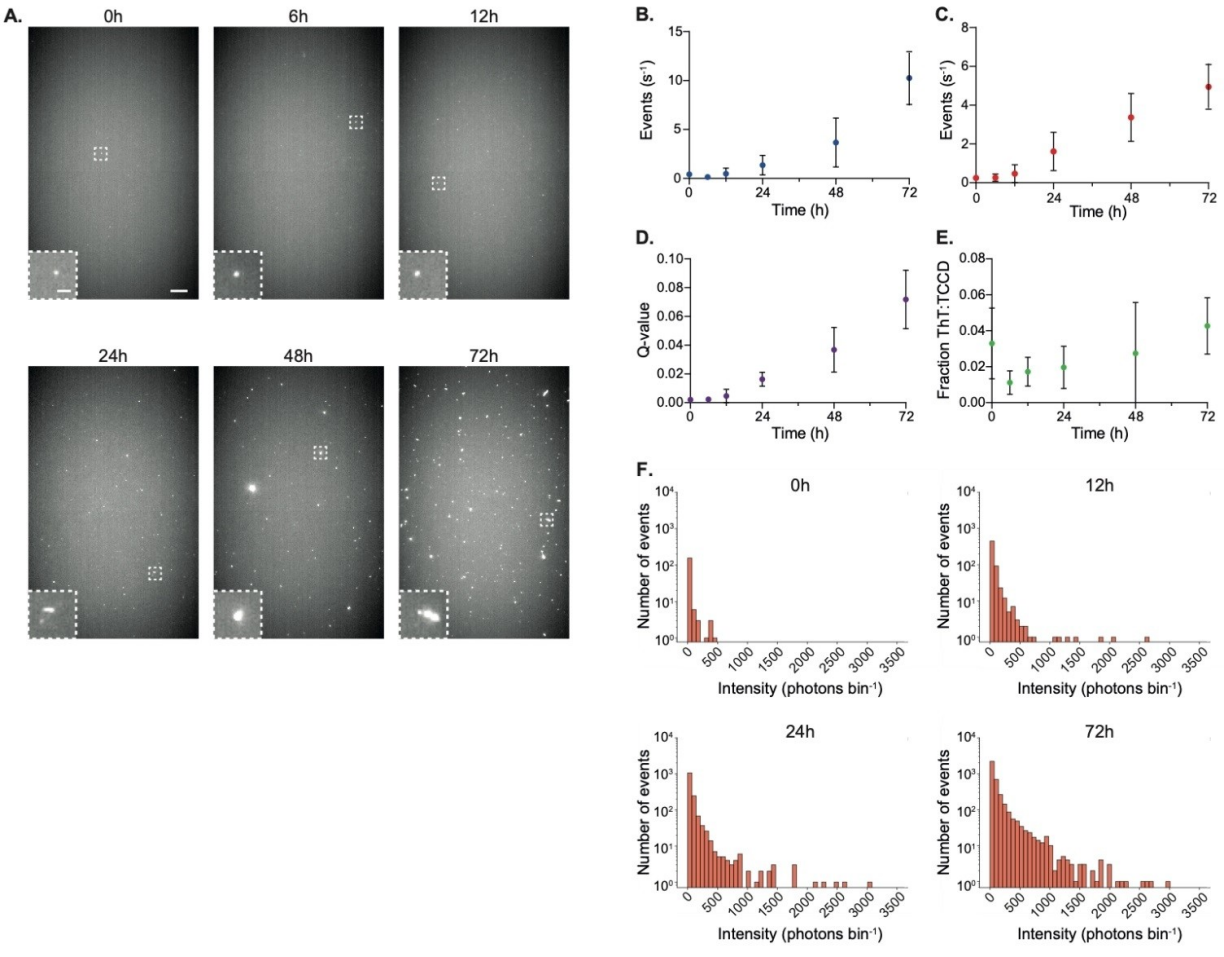
The TCCD approach effectively tracks the aggregation of unlabeled α‐syn. a) Representative SAVE images of 500 nM α‐syn aggregates collected at multiple timepoints. Scale bars are 5 μm and 1 μm in length for full field of view and insets respectively. b) ThT detection of 500 nM α‐syn aggregates using the single‐molecule confocal microscope. c) TCCD event rate from a 10 nM α‐syn sample. d) Association quotient for each timepoint. e) The fraction of TCCD events which were also ThT active. f) Intensity distributions (summed from both channels) from aggregates detected at different timepoints. All data in b–e plotted as mean±SD, *n*=3.

Samples of α‐syn subjected to conditions favoring aggregation were taken at several timepoints between 0 and 72 hours, and observed using SAVE imaging and single‐molecule confocal microscopy with ThT, and with TCCD. In the first 12 hours of the aggregation, negligible numbers of aggregates were visible using SAVE imaging (Figure 4a); however, the number, size and intensity of the aggregates visibly increased over the next 60 hours. This trend was also observed for ThT‐detection on the single‐molecule confocal microscope (Figure 4b). Whereas ThT is only able to detect aggregates that have an extended ß‐sheet structure, our approach should be able to detect any complex formed from multiple α‐syn monomers, including both oligomers and fibrils. For TCCD, both the event rate (Figure 4c) and association quotient (Figure 4d) increased over time similarly to ThT detection; however, the samples for TCCD were diluted to a 50× lower concentration to enable single‐molecule detection.

Comparison of the event rates therefore shows that our approach is able to detect ≈20x more species than possible with ThT. A comparison of the event rates for each detection method revealed that <5% of aggregates detected by TCCD were also ThT‐active (Figure 4e), highlighting the vast improvement in sensitivity afforded by TCCD compared with using amyloid‐binding dyes. Additionally, the total intensity of each aggregate detected could also be determined, and while their absolute size cannot be determined accurately due to the variation in antibody labeling stoichiometry (Figures S2 and S3), and the stochastic distribution of photon counts in single‐molecule confocal microscopy,^[31]^ when binned into histograms, there was a noticeable shift of the population to higher intensities at later timepoints (Figure 4f). This indicates that a higher number of antibodies were bound to each aggregate, which is to be expected as their size increased with time. For more accurate sizing, an alternative approach utilizing fluorescence cross‐correlation spectroscopy could be applied to the dual‐labeled aggregates to determine their hydrodynamic radii.^[32]^

These data demonstrate our TCCD approach as an effective method to track the self‐assembly of unlabeled α‐syn into higher order species. Furthermore, by comparing with ThT‐detection, it also allows the determination of the proportion of aggregates that have acquired extended β‐sheet structure.

### TCCD measurements in a biological sample

**Figure 5:**
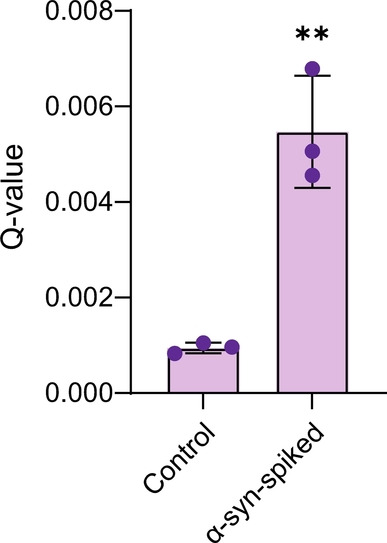
Detection of α‐syn aggregates in human CSF. CSF was spiked with 500 pM sonicated α‐syn aggregates subjected to aggregating conditions for 120 h. To account for increased background fluorescence events thresholds were increased to 11 and 19 photons bin−1 for the blue and red channel, respectively. Mean±SD, *n*=3, unpaired student t test (** *P*<0.005).

Finally, to verify that TCCD can be used to accurately detect unlabeled α‐syn structures in a complex biological mixture, 500 pM of aggregated recombinant α‐syn was spiked into human cerebrospinal fluid (CSF), which contains multiple proteins and other macromolecules. Under these conditions, the oligomer concentration is approximately 1–5 % of the total monomer concentration, which is close to approximated physiological levels of oligomers in PD CSF (≈10 pM).[^33^, ^34^] The significant increase in *Q‐*value when compared with unspiked CSF demonstrates that our technique can robustly detect picomolar levels of α‐syn aggregates in a complex biological sample (Figure 5).

## Conclusion

Amyloid‐binding dyes have been used extensively to study the aggregation kinetics of α‐syn at the single‐molecule level, as well as probe clinical samples for the presence of protein aggregates.[^16^, ^19^, ^20^] Their lack of specificity, however, can be problematic, given the high prevalence of ThT‐active species in clinically healthy individuals.^[16]^ The TCCD single‐molecule confocal technique developed here not only allows for specific detection of α‐syn oligomers but also provides >20‐fold greater sensitivity when compared with ThT detection. Moreover, using antibody detection removes the need for covalently bound fluorescent labels, enabling the aggregation kinetics of α‐syn to be studied without the potentially pejorative effects of the dye. Our approach could therefore be used for monitoring the inhibition of α‐syn aggregation by various compounds. Finally, the ability to specifically observe higher order conformers of unlabeled α‐syn in a complex biological mixture raises the possibility of using this method to measure α‐syn oligomeric load in clinical samples. This in turn could potentially be used as an indicator of disease.

## Conflict of interest

The authors declare no conflict of interest.

1

## Supporting information

As a service to our authors and readers, this journal provides supporting information supplied by the authors. Such materials are peer reviewed and may be re‐organized for online delivery, but are not copy‐edited or typeset. Technical support issues arising from supporting information (other than missing files) should be addressed to the authors.

Supporting Information

## Data Availability

The data that support the findings of this study are availablefrom the corresponding author upon reasonable request.
