# Supplementary material for: Single‐Molecule Two‐Color Coincidence Detection of Unlabeled alpha‐Synuclein Aggregates
Source: Angew Chem Int Ed Engl. 2023 Feb 28;62(15):e202216771. doi: 10.1002/anie.202216771 (PMC10946743; doi:10.1002/anie.202216771)
Supplement: Supplementary file 1 — Supporting Information [file ANIE-62-0-s001.pdf]

Angewandte Chemie
Eine Zeitschrift der Gesellschaft Deutscher Chemiker

Supporting Information

Single-Molecule Two-Color Coincidence Detection of Unlabeled alpha-Synuclein Aggregates

*A. Chappard, C. Leighton, R. S. Saleeb, K. Jeacock, S. R. Ball, K. Morris, O. Kantelberg, J.-E. Lee, E. Zacco, A. Pastore, M. Sunde, D. J. Clarke, P. Downey, T. Kunath, M. H. Horrocks**

Experimental Procedures

α-Synuclein expression and purification
Wild-type (WT) α-syn was purified from *E.coli* following the protocol described by Hoyer et al.[1] Following purification, the protein was divided into aliquots having concentrations of 200-300 μM in 25 mM Tris buffer (pH 7.4) and 100 mM NaCl, before being flash-frozen in liquid nitrogen and stored at −80 °C.

Mass spectrometry
Intact protein mass was determined by liquid chromatography-mass spectrometry (LC-MS) on a quadrupole time-of-flight instrument, Synapt G2S (Waters Corp.). A reverse-phase C4 analytical column was used for chromatographic separation of proteins and salts in the sample, with an organic gradient of acetonitrile and 0.2% formic acid. Mass deconvolution was performed using the Maximum Entropy (MaxEnt) tool on MassLynx v4.1 (Waters Corp.).

Aggregation of α-syn
To remove pre-formed aggregates, α-syn monomer was ultracentrifuged (Beckman OptimaMax) at 90,000 g for 1h at 4°C. The pellet was discarded, and the protein concentration of the supernatant was determined from the absorbance at 275 nm using an extinction coefficient of 5,600 $M^{-1}cm^{-1}$. The protein was diluted in 25 mM Tris (pH 7.4), 100 mM NaCl, 0.01% (w/v) NaN_3 to a total protein concentration of 70 μM. The buffer was freshly prepared before each experiment and passed through a 0.02 μm syringe filter (Anotop, Whatman) to remove insoluble contaminants. The aggregation mixture was kept in DNA LoBind microcentrifuge tubes (Eppendorf) and left shaking (200 r.p.m) at 37°C in an incubator (StuartScientfic) for the duration of the experiment. Aliquots were taken at a series of timepoints over the incubation period and were immediately snap-frozen in liquid nitrogen. Timepoints were stored at -80°C until required for analysis.

Antibody affinity
Interaction kinetics were determined by using surface plasmon resonance (SPR) technology on a Biacore T200 instrument. Purified recombinant human α-syn fibrils were immobilized on flow cells of a CM5 chip surface using amine-coupling chemistry. Human α-syn fibrils were prepared in 10 mM NaAc, pH 3.5, and immobilized on separate flow cell surfaces to reach an immobilization level of about 40 response units (RU) at a flow rate of 10 ml min^{-1}. The buffer HBS-EP+ (GE healthcare Bio-Sciences AB) was used as running buffer for both ligand immobilization and kinetics assay. The binding of anti-alpha synuclein monoclonal mouse IgG1 was then measured. The monoclonal IgG was injected at 7 different concentrations from 800 nM to 0.195 nM over the 3 flow cells with a contact time of 3 mins and a disassociation time of 30 mins, at a flow rate of 100 ml min^{-1}. The surface was regenerated by one injection of 50 mM HCl for 90 s at 10 ml/min, and another injection of 50 mM HCl for 60 s at 10 ml min^{-1}. The data were analyzed using the Biacore T200 evaluation software (version 3.0) using the bivalent analyte model with assumed no bulk contribution (RI=0) and global R_{max} for IgG format, and 1:1 model with flexible bulk contribution (local RI) and global R_{max}. The binding kinetics are summarized in table S1.

Binding constants to human α-syn fibrils		
K_a ($M^{-1}s^{-1}$)	K_d (s^{-1})	K_D (nM)
5.7 E+05	1.8E-05	0.03

Table S1. Binding kinetics of the antibody used in this study to α-syn fibrils, determined by SPR.

Antibody labeling
The antibody used in this study was provided by UCB Biopharma. 100 μg of the antibody was labeled with either AF488 or AF647 using antibody labelling kits according to the manufacturer's instructions (A20181, A20186, Thermofisher). Briefly, 100 μg of antibody was added to 0.1 M sodium bicarbonate, before incubation with the required dye molecule modified with a succinimidyl ester moiety. The dye was left to react with primary amines on the antibody for 1h in the dark at RT. Unbound dye was removed using a micro spin column before determining concentration by measuring absorbance at 280 nm for protein and 494 nm and 650 nm for AF488 and AF647 dyes, respectively. The antibodies were then stored at 4°C until required. Photobleaching step counting was performed to determine the labeling stoichiometry of the antibodies (Figure S2).

Preparation of Thioflavin-T

Initial stock solution (~10 mM) was prepared by dissolving ThT (ab120751, Abcam) into neat Ethanol. This was then used to make a ~200 µM stock in 25 mM Tris (pH 7.4), 100 mM NaCl. This stock was made fresh before use and passed through a 0.02 µm syringe filter (Anotop, Whatman) to remove insoluble contaminants. The exact concentration of this solution was determined by measuring absorbance at 412 nm using an extinction coefficient of 36,000 $M^{-1}cm^{-1}$

Microfluidic device

The microfluidic device design has been described previously[2], and consists of a single channel (width = 100 µm, height = 25 µm, length = 1 cm). Microfluidic devices were fabricated using standard soft-lithography techniques into polydimethylsiloxane (PDMS; Dow Corning) with SU-8 photoresist on silicon masters, as described previously[3]. The channels were oxygen plasma-bonded to glass coverslips (V.W.R., thickness = 1) to create sealed devices. Each device was inspected on a white-light microscope (Nikon TE2000-U), and only those without dust or aberrations were used.

Single-molecule confocal measurements

For all single-molecule measurements of recombinant aggregates, 70 µM aliquots were recovered from -80°C and thawed on ice. For ThT measurements, the aliquots were diluted to a concentration of 500 nM into 5 µM ThT in filtered 25 mM Tris (pH 7.4) with 100 mM NaCl. All stated concentrations for aggregated protein are given as the monomer equivalent concentration. For TCCD measurements, the aliquots were diluted at 10x stated concentration into a solution of 200 pM AF488-tagged antibody and 200 pM AF647-tagged antibody in filtered 25 mM Tris (pH 7.4) with 100 mM NaCl. These were incubated for 10 minutes before diluting 10-fold into 25 mM Tris (pH 7.4) with 100 mM NaCl. For α-syn aggregation experiments, 1mg/ml BSA (Sigma-Aldrich, 9048468) was added to the 1000x antibody stock to minimize adsorption to the microcentrifuge tube. The samples were subsequently loaded into the microfluidic inlet port using 200 µl gel-loading tip (Fisher scientific, 11927734). Diluted protein samples were withdrawn through a single-channel microfluidic device at a flow velocity of 1 cm s^{-1} to a syringe (1 mL, Braun Injekt, 9166017V)) via Fine Bore Polyethylene Tubing (0.38 mm inner-diameter, 1.09 mm outer diameter; Smiths Medical International, Hythe, Kent, UK). Flow control was achieved using a syringe pump (Kd scientific, 78,8101), and the device was mounted on the microscope stage.

All confocal microscopy measurements were performed on a custom built single-molecule confocal microscope described previously[4]. Briefly, an Oxxius L6Cc laser combiner (Oxxius, France) containing Gaussian laser beams of three wave lengths 405 nm (LBX-405-100-CSB-OE, Oxxius), 488 nm (LBX-405-100-CSB-OE, Oxxius) and 638 nm (LBX-405-100-CSB-OE, Oxxius) was used as a laser source. Laser beams were overlapped within a 0.13 NA optical fiber (Thorlabs) and collimated by a reflective collimator (RC08APC-P01, Thorlabs), and directed through the back-port of an inverted microscope (Nikon TE2000-U). A dichroic mirror (DI03-R405/488/561/635, Semrock) reflected the beam through an oil-immersion objective lens (Nikon CFI Plan Apochromat VC 100x Oil, NA 1.4, W.D 0.13 mm) that focused the light to a diffraction-limited confocal spot. The emitted fluorescence was collected by the same objective lens and was passed through the same dichroic mirror, before being focused by a tube lens within the microscope body through a 50 µm pinhole (Thorlabs). A second dichroic mirror (585DRLP, Omega Filters) was used to separate the fluorescence from the two different fluorophores. The longer wavelength passed through the dichroic mirror and was focused by a lens (Plano apoconvex, focal length = 50 mm, Thorlabs) through a filter set (long-pass: LP02-647RU-25, Semrock, and band-pass: FF01-432/515/595/730-25, Semrock) onto an Avalanche Photodiode (APD) detector (PerkinElmer). The shorter wavelengths were reflected and focused through a second filter set (long-pass: BLP01-488R-25 and band-pass: FF01-525/30-25, Semrock) onto a second APD. Outputs from the APDs were connected to a USB data acquisition card (USB-CTR04, Measurement Computing), which counted the signals and combined them into time-bins of 100 µs, the expected residence time of the molecules in the confocal volume. For ThT measurements, the sample was excited with 405 nm irradiation (4.4 mW at the back-port of the microscope), and for TCCD measurements, 488 nm and 647 nm irradiation was used (6.35 mW and 0.6 mW at the back-port, respectively).

SAVE imaging

For SAVE imaging, 22 x 40 mm 0.1 mm thickness slides (VWR, 6310135) were plasma cleaned (Diener Zepto plasma cleaner) with an argon ion plasma for 3 x 20-minute cycles to remove organic material. The slides were then affixed with 9x9 mm well gaskets (Biorad, SLF0201), and 50 µl PLL (Sigma-Aldrich, 25988-63-0) was added, incubated for 30 minutes, and subsequently washed off three times with 0.02 µm-filtered buffer. 70 µM aliquots were recovered from -80°C and thawed on ice before being diluted to a concentration of 500 nM into 5 µM ThT in 0.02 µm-filtered 25 mM Tris (pH 7.4) with 100 mM NaCl. Imaging was performed on a commercial total internal reflection fluorescence microscope (TIRF, Oxford Nanoimager). The TIR angle was set at 53°. A 5 x 5 imaging grid

spaced 200 μm apart was measured and was automated to prevent user bias. 40% 405 nm laser power was used for imaging, and images were recorded at 20 frames/s for 100 frames.

Preparation of TDP-43 aggregates
The two RNA recognition motifs (RRMs) of TDP-43 were expressed and purified in tandem (referred to as RRM1-2), as described previously[5]. Purified RRM1-2 samples stored at -80 °C were rapidly defrosted and diluted to 20 μM in a high salt buffer (10 mM potassium phosphate buffer pH 7.2, 150 mM KCl). Constructs were subsequently centrifuged at 90,000 g for 1 h to remove any aggregated protein. The final protein concentration was assessed by absorbance at 280 nm and adjusted to 15 μM. Protein aggregation was carried out at 37 °C under non-shaking conditions, and aliquots were taken at 48h and flash frozen in liquid nitrogen.

Preparation of Tau aggregates
Tau4R monomer stored at -80°C in 10 mM ammonium acetate, pH 8.5, was rapidly defrosted and diluted to 20 μM in SSPE buffer supplemented with 2 μM heparin and 0.01% NaN_3. Protein aggregation was carried out at 37 °C under non-shaking conditions, and aliquots were taken at 2 weeks and flash frozen in liquid nitrogen.

Preparation of amyloid-β aggregates
Aβ monomer (AS-20276) was diluted to 2 μM in SSPE buffer and protein aggregation was carried out at 37 °C under non-shaking conditions, and aliquots were taken at 48h and flash frozen in liquid nitrogen.

Cerebrospinal fluid
The CSF sample used in this study was collected for a previous biomarker study[6], and was obtained from UCL Queen Square Institute of Neurology. Informed consent was obtained from all subjects, including access to their clinical data. No additional ethics approval was required for our use of the CSF after obtaining it from the UCL Queen Square Institute of Neurology. CSF was collected by lumbar puncture from a healthy control. The sample used was classified as a non-PD, normal healthy individual according to clinical observation.

Data analysis
A custom-written Python code was used to read and analyse TCCD data (10.5281/zenodo.7109861). Photon bursts were first corrected for autofluorescence, given by the average signal intensity in absence of fluorescent molecules and cross-talk between both channels. 3% of signal intensity in the red channel was determined to be cross talk from the blue channel, whilst crosstalk in the other direction was negligible.

Blue channel intensity correction:

$$IB = B - AB$$

Where IB is the modified intensity in the blue channel, B is the original intensity and AB is the autofluorescence in the blue channel

Red channel intensity correction:

$$IR = R - AR - (C \times B)$$

Where IR is the modified intensity in the red channel, R is the original intensity, AR is the autofluorescence in the red channel and C is the cross-talk from the blue channel.

The number of photons in a time bin required to count as an event above the background was determined according to previously established threshold selection approaches (SI Figure 1B)[7]. Simultaneous events in both channels were assessed using the AND criterion[8], which accepts only those signals for which both the blue-excited channel AND the red-excited channel are above their respective threshold value. To account for coincident events that could occur due to chance, a desynchronisation approach was used[7]. Time-bins in the red channel were randomly shuffled and the number of simultaneous events in the two channels above the threshold was re-calculated, the resulting number of events were classified as chance events. The fraction of coincident events, with respect to chance was then calculated (SI Figure 1A).

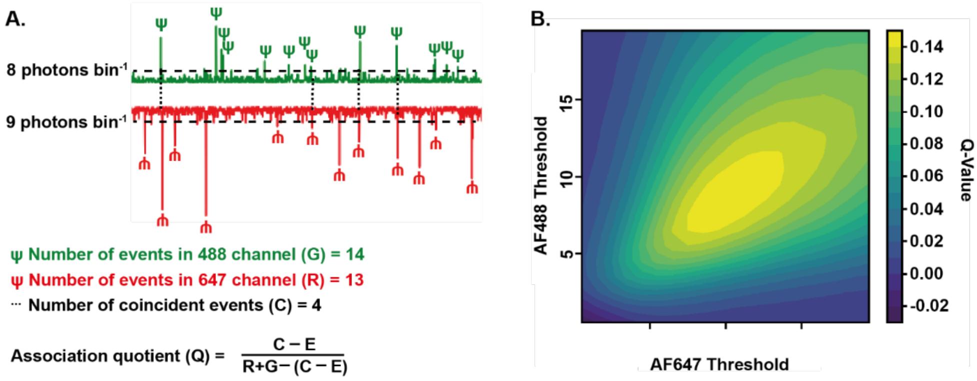

Figure S1. (A) Schematic representation of how the Q-value is calculated from single-molecule, TCCD data. A and B are the number of events in the two channels, C is the number of coincident events and E is the number of events which occur by chance. (B) Contour map showing how the Q-value varies as a function of the applied event threshold in each channel. Thresholds of 8 and 9 photons bin^{-1} for the blue and red channels, respectively, were applied to all data unless otherwise stated.

Determination of antibody labeling stoichiometry

The α-syn antibody was labeled by conjugating its primary amines to NHS-ester modified fluorophores (either AF488 or AF647). The availability of multiple primary amines on lysine residues, coupled with the stochastic nature of the labeling reaction, results in a variation in the antibody labeling efficiency. This affects both the detection efficiency of the antibodies, and the size determination of α-syn aggregates. We used photobleaching step analysis to quantify the labeling stoichiometry of the antibodies.

Borosilicate glass coverslips 20 x 40mm were cleaned using an argon plasma cleaner for 1 hour to remove any fluorescent residues. 9 x 9 mm well gaskets were affixed to the glass coverslips, before 400 pM of AF488- and AF647-antibodies were incubated on the surface and allowed to adsorb for 5 minutes. They were then imaged on the TIRF microscope (Oxford Nanoimager) for 200 frames per image and a total of 50 fields of view were captured. The TIRF angle was set at 53.5^{o}, the exposure time was 50 ms, and the 488 nm and 647 nm lasers were set at power levels of 12% and 14%, respectfully. Data were analysed using a custom MATLAB script (10.5281/zenodo.6473021). A prominence value of 75 and 100 was used to select maxima in the first frame for the 488 and 647 channels, respectively, and only maxima detected in the first frame were followed for the duration of the imaging to filter out "blinking" events. Intensity profiles were generated for each spot (SI Figure 2), and then filtered using Chung-Kennedy filter with a sensitivity factor of 50 and a filter window size of 10. A peak threshold, minimum differential of the filtered intensity trajectory for the step to count, was set at 35 and 20 for 488 nm and 647 nm channels respectively. A confidence threshold of a step having occurred was set at 0.1. The stoichiometry histograms are shown in SI Figure 3A, and each antibody had an average of 1.99 fluorophores for AF488 and 2.04 fluorophores for AF647. We were unable to rule out the presence of unlabeled antibody using this approach. Due to differences in labelling efficiency, antibody concentration was normalised to the number of fluorescent events generated by singularly labeled DNA strands (SI Figure 3B).

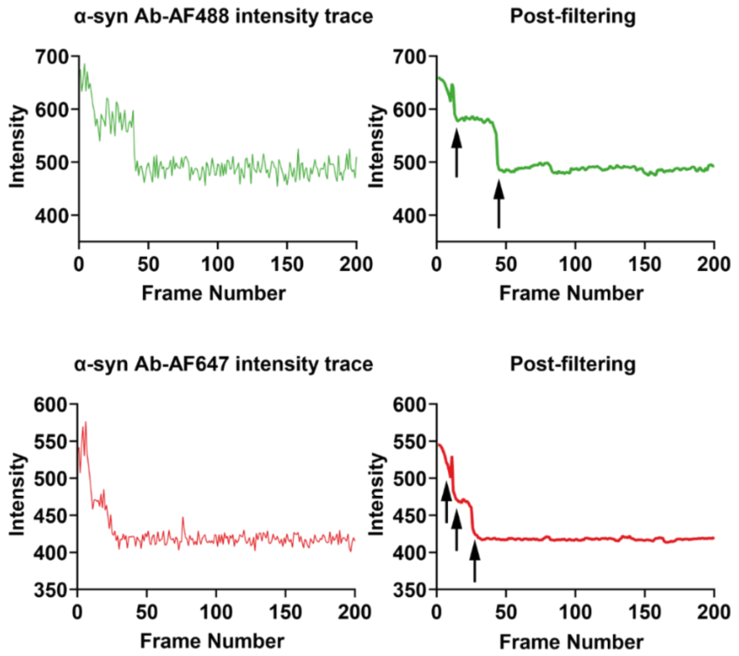

Figure S2. Example intensity profiles for AF488 and AF647 tagged antibodies. Post filtering enables the detection and counting of photobleaching steps which were used to determine the labeling stoichiometry of the antibodies.

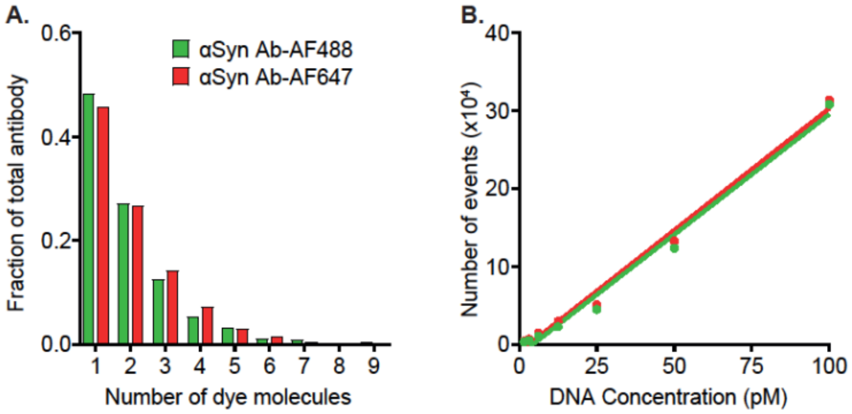

Figure S3. (A) Labeling stoichiometry histograms for AF488- and AF647-tagged antibodies. The number of dye molecules per antibody was determined by counting photobleaching steps. (B) A range of concentrations of AF488- or AF647-labeled DNA was measured on the single-molecule confocal microscope, and the event rate determined. The data were fit to a straight line (AF488 - green, R^2 = 0.99; AF647 - red, R^2 = 0.99) to determine the relationship between the event rate and the concentration of label, and this was used to determine the labeled antibody concentrations.

Determination of optimal antibody concentration

Selection of the optimum antibody concentration for single-molecule detection of α-syn aggregates is not trivial. A low concentration will result in too few oligomers being labeled and detected, whereas a high concentration will lead to multiple occupancy of the confocal volume and the inability to resolve individual molecules. To determine the optimum concentration, a sample of wild-type (WT) α-syn was aggregated for 24 hours and diluted to 100 pM,

before being incubated with a range of concentrations of labeled antibody and detected on the single-molecule confocal microscope with dual color excitation (Figure S4a). The product of the event rate and Q against the varying antibody concentration was used to determine an optimal antibody concentration of 40 pM (Figure S4b).

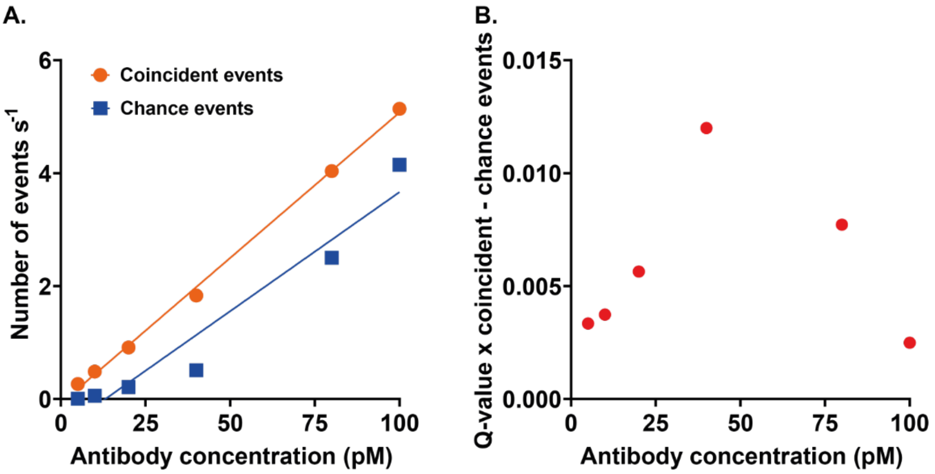

Figure S4. Optimal antibody concentration for TCCD detection. Determining optimal antibody concentration for TCCD using a fixed sample of 100 pM α-syn subjected to aggregating conditions for 24h. (A) The effect of antibody concentration on the coincidence (R^2 = 0.99) and chance event rate (R^2 = 0.94). (B) The product of the association quotient and event rate increases from 5 to 40 pM, before decreasing. Data were acquired for 10 minutes for each sample and event thresholds were set at 8 and 9 photons bin^{-1} for green and red channels, respectively.

Mass spectrometry analysis of recombinant protein

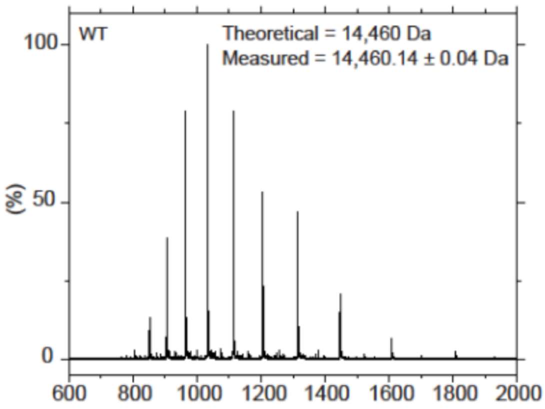

Figure S5: Mass-spectrum of purified α-syn.

Non-specific antibody binding

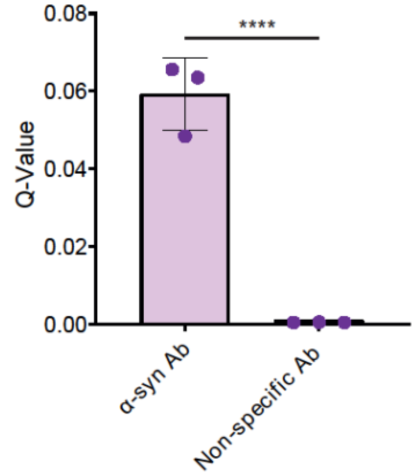

Figure S6: TCCD analysis of 10 nM α-syn subjected to aggregating conditions for 24h using α-syn antibody or non-specific secondary antibodies labelled with AF488 or AF647. Mean ± SD, n = 3, **** P < 0.001 unpaired student t-test.

Supplementary References

[1] W. Hoyer, T. Antony, D. Cherny, G. Heim, T. M. Jovin, V. Subramaniam, *Journal of Molecular Biology* **2002**, *322*, 383–393.
[2] M. H. Horrocks, L. Tosatto, A. J. Dear, G. A. Garcia, M. Iljina, N. Cremades, M. Dalla Serra, T. P. J. Knowles, C. M. Dobson, D. Klenerman, *Anal. Chem.* **2015**, *87*, 8818–8826.
[3] M. H. Horrocks, H. Li, J. Shim, R. T. Ranasinghe, R. W. Clarke, W. T. S. Huck, C. Abell, D. Klenerman, *Anal. Chem.* **2012**, *84*, 179–185.
[4] M. L. Choi, A. Chappard, B. P. Singh, C. Maclachlan, M. Rodrigues, E. I. Fedotova, A. V. Berezhnov, S. De, C. J. Peddie, D. Athauda, G. S. Virdi, W. Zhang, J. R. Evans, A. I. Wernick, Z. S. Zanjani, P. R. Angelova, N. Esteras, A. Y. Vinokurov, K. Morris, K. Jeacock, L. Tosatto, D. Little, P. Gissen, D. J. Clarke, T. Kunath, L. Collinson, D. Klenerman, A. Y. Abramov, M. H. Horrocks, S. Gandhi, *Nat Neurosci* **2022**, *25*, 1134–1148.
[5] E. Zacco, O. Kantelberg, E. Milanetti, A. Armaos, F. P. Panei, J. Gregory, K. Jeacock, D. J. Clarke, S. Chandran, G. Ruocco, S. Gustincich, M. H. Horrocks, A. Pastore, G. G. Tartaglia, *Nat Commun* **2022**, *13*, 3306.
[6] N. K. Magdalinou, R. W. Paterson, J. M. Schott, N. C. Fox, C. Mummery, K. Blennow, K. Bhatia, H. R. Morris, P. Giunti, T. T. Warner, R. de Silva, A. J. Lees, H. Zetterberg, *J Neurol Neurosurg Psychiatry* **2015**, *86*, 1240–1247.
[7] Richard W. Clarke, Angel Orte, D. and Klenerman*, *Analytical Chemistry* **2007**, *79*, 2771–2777.
[8] Angel Orte, Richard Clarke, Shankar Balasubramanian, D. and Klenerman*, *Analytical Chemistry* **2006**, *78*, 7707–7715.
